# Functional and three-dimensional radiographic outcomes after open reduction and internal fixation of condylar head fractures using magnesium alloy cannulated screws – a retrospective long-term follow-up

**DOI:** 10.1007/s00784-025-06585-x

**Published:** 2025-09-29

**Authors:** Henry Leonhardt, Jan Bernard Matschke, Philipp Sembdner, Alexander Seidler, Niall M. H. McLeod, Christian Bräuer, Adrian Franke

**Affiliations:** 1 Consultant for Oral and Maxillofacial Surgery, Department of Oral and Maxillofacial Surgery, University Hospital Carl Gustav Carus Dresden, Technische Universität Dresden, Fetscherstraße 74, 01307 Dresden, Germany; 2 Intern for Oral and Maxillofacial Surgery, Department of Oral and Maxillofacial Surgery, University Hospital Carl Gustav Carus Dresden, Technische Universität Dresden, Fetscherstraße 74, 01307 Dresden, Germany; 3 Chair of Virtual Product Development, Institute of Machine Elements and Machine Design, TU Dresden, 01062 Dresden, Germany; 4 Consultant Oral & Maxillofacial Surgeon at University Hospitals Coventry & Warwickshire NHS Trust, Clifford Bridge Rd, Coventry, CV2 2DX UK; 5 Consultant for Oral and Maxillofacial Surgery, Department of Oral, Maxillofacial and Facial Plastic Surgery, Rostock University Medical Center, Schillingallee 35, D-18057 Rostock, Germany; 6 Klinik und Poliklinik für Mund-, Kiefer- und Gesichtschirurgie, Universitätsklinikum Carl Gustav Carus an der Technischen Universität Dresden, 01304 Dresden, Germany

**Keywords:** Magnesium, Biodegradable implants, Mandibular condyle, Internal fracture fixation, Three-dimensional imaging, Mandibular fractures

## Abstract

**Objectives:**

Condylar head fractures are common in facial trauma and can result in significantly impaired mandibular function. Open Reduction and internal fixation overall produce better functional outcomes than closed treatment. Multiple methods of fixation have been proposed, including the use of biodegradable magnesium-based cannulated bone screws. This study aims to investigate the long-term efficacy of this fixation method using three-dimensional radiographic imaging and clinical outcomes.

**Materials and methods:**

This retrospective cohort study of a single centre included patients who underwent surgical intervention for a condylar head fracture using a cannulated magnesium lag screw. Clinical parameters and three-dimensional radiographic scans were collected during follow-up, and virtual model analysis was conducted after segmentation.

**Results:**

Fifty-eight patients received an osteosynthesis with a magnesium alloy implant. The functional results of the final assessment were excellent. Significant changes in volume, surface area, and signs of condylar remodelling were observed during the healing process. Complication rates were low. No implant had to be removed.

**Conclusions:**

Cannulated compression screws using biodegradable magnesium-based alloys show good clinical results despite reduced condylar volume and surface area, as well as significant proof of three-dimensional incongruency of healing mandibular condyles. No patient complained of subjective problems connected to the resorption process, and no mechanical failure prior to fracture healing was recorded. Overall, this method verifies excellent long-term results.

**Clinical relevance:**

Surgical implants comprised of magnesium alloys provide adequate fixation of fractures of the condylar head with excellent clinical results without the need for a second intervention to remove the implant.

**Supplementary Information:**

The online version contains supplementary material available at 10.1007/s00784-025-06585-x.

## Highlights

- magnesium alloy implant for osteosynthesis of mandibular condylar head fractures.

- good clinical long-term results despite morphological changes in the condylar head.

- novelty analysis method quantifying three-dimensional morphological changes.

**Supplementary Information:**

The online version contains supplementary material available at 10.1007/s00784-025-06585-x.

## Introduction

In traumatology of the facial skeleton, the mandible represents a considerable proportion [1–3] of fractures seen, with the condylar head being one of the most affected subsites [4–6]. This site is particularly special as it forms part of the temporomandibular joint, a complex structural unit which forms the articulation between the mandible and cranium [7]. Condylar head fractures (CHF) may result in impaired occlusion and deflection or impaired mobility of the mandible, perhaps even leading to inadequate food intake.

The treatment of CHF can be divided into conservative, closed and open reduction and internal fixation (ORIF) methods. Closed treatment necessitates immobilising the mandible, typically with intermaxillary wiring for ten days with subsequent functional elastics. With internal fixation, the jaws are sometimes generally immobilised intraoperatively or a few days after the operation. This reduction in the time of immobilisation of the temporomandibular joint significantly reduces treatment times and allows early rehabilitation with the recovery of function.

Surgical treatment of CHF is strongly recommended when associated with a loss of the height of the ascending mandibular branch. This situation is specifically present in type B and C fractures [8, 9] or type p fractures according to the current AOCMF classification [10, 11].

Many techniques for osteosynthesis have been suggested for the surgical treatment of this complex anatomical structure in the past. They range from mini- and microplates [12–14] to mono- and bicortical titanium screws [15–17] and small fragment screws [18, 19], to biodegradable polylactide pins [20, 21] and magnesium-based cannulated screws [22, 23]. It is not easy to compare these innumerable techniques of osteosynthesis, and research endeavours must emphasise a combination of imaging, clinical-functional, instrumental, and experimental approaches [24].

We have previously used titanium-based cannulated screws, but in a small number of cases, “migration” of the screw through or remodelling of the condylar head led to penetration through the articular surface into the joint space, necessitating the removal of the osteosynthesis material [25]. Screws with magnesium-based alloys have been used in the same surgical procedure and have shown promising clinical results [26], but there have been, to date, no long-term follow-up studies covering the biodegradation process and true three-dimensional remodelling.

## Materials and methods

### Study design

Adhering to the STROBE (strengthening the reporting of observational studies in epidemiology) guidelines, the study was planned and conducted as a retrospective cohort study performed at a single centre enrolling patients with a condylar head fracture treated at the Clinic for Oral and Maxillofacial Surgery of the Carl Gustav Carus University Hospital in Dresden between May 2016 and September 2022 using a magnesium-based cannulated compression screw (Mg-CS). Inclusion criteria were legal consent of the patient or legal guardian for the surgical intervention, the use of a magnesium-based cannulated compression screw (Mg-CS) for osteosynthesis of the condylar head, and regular follow-ups with at least two postoperative radiographs during follow-up. Exclusion criteria were failure of follow-up and denial of participation in the study.

The study was performed in line with the principles of the Declaration of Helsinki. The Ethics and Institutional Review Board of the University Hospital Carl Gustav Carus at the Technical University of Dresden (institutional review board number IRB00001473) registered at the Office for Human Research Protections (IORG0001076) approved the study (internal ethics committee ID number: EK 453102016).

## Implant

**Fig. 1:**
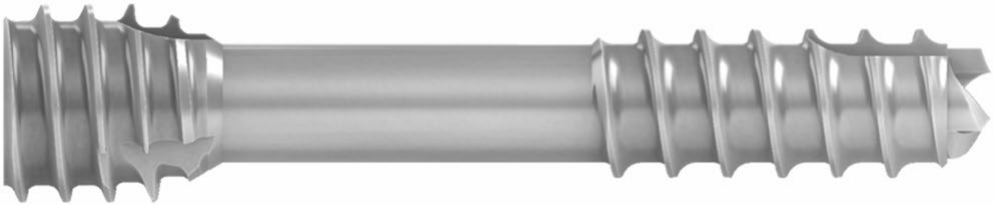
A detailed picture of the cannulated Magnezix CS 2.7 screw. The differing thread pitches of the tip and the head, which allow for fragment compression, can be readily observed. Syntellix AG (Hannover, Germany) consented to the publication of the image

The magnesium alloy headless bone screw Magnezix^®^ CS 2.7 lag screw from Syntellix AG (Hannover, Germany; article number 1027.0xx) was used to fixate the reduced fractures after Kirschner wire stabilisation. The hollow design of the screws allows fixation of the reduced fracture in an envelope style, placing the implant precisely at the intended position. The differing thread pitches of the tip and the head exert compression on the fracture after gripping each thread to its fragment, resulting in high osteosynthesis stability and promoting bone healing (Fig. 1).

## Operation method

**Fig. 2:**
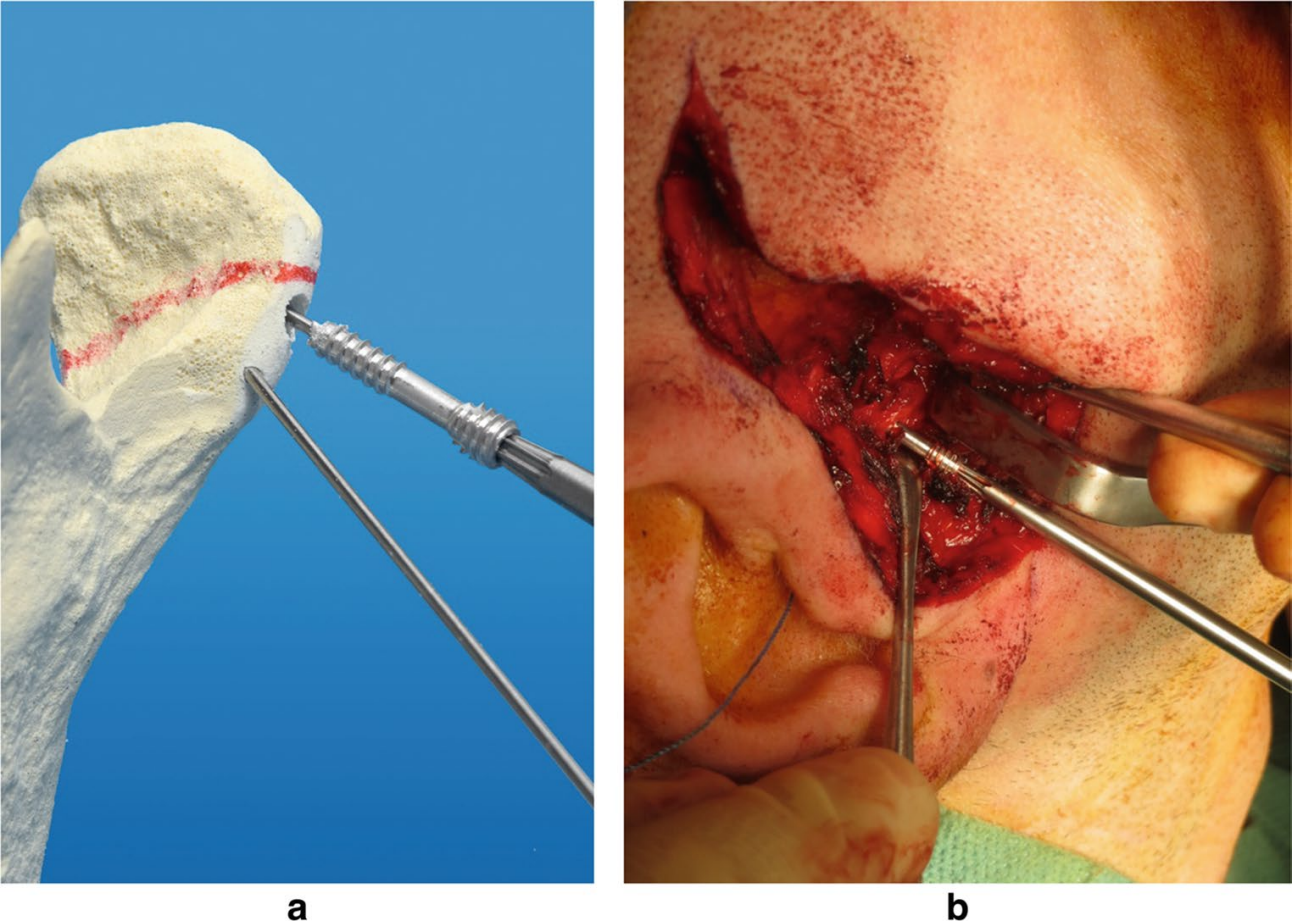
Depiction of the operation method applied. (**A**) Two K-wires are inserted in a model of a condylar head fracture on the left side. The Mg-CS is inserted into the prepared drill hole over one of the K-wires. (**B**) Intraoperative picture of the insertion of the Mg-CS that is inserted into the reduced condylar head fracture on the right side

The Mg-CS is inserted similarly to a typical headless bone screw. The preauricular approach is used to reveal every fracture. The anteromedially displaced condylar head fragment is mobilised to reduce the fracture, and one or two 0.9 mm K-wires are inserted to stabilise the condylar head fragment temporarily. A cannulated drill is utilised to expand the hole in the condylar neck. Close attention is paid to not extend the drill hole into the proximal fragment of the condylar head to increase the hold of the tip thread of the Mg-CS in the condylar head fragment. The Mg-CS is then inserted over the K-wire, and compression over the fracture is provided at the end of the insertion, when the different threads of the tip and the head of the screw grip its surrounding bone. The Mg-CS must be countersunk entirely into the bone to prevent lateral contact of the alloy with the soft tissue (Fig. 2). To assure high patient safety, at least one experienced consultant in oral and maxillofacial surgery for traumatology performed the surgery. After surgery, intermaxillary fixation employing elastics was applied for two days to avoid joint effusion [22, 26].

### Follow-up

Time clusters were used for routine follow-up examinations; thus, follow-up times varied from 0 to 50 days in Cluster 1, 51 to 100 days in Cluster 2, 101 to 200 days in Cluster 3, 201 to 400 days in Cluster 4, and 401 days and beyond in Cluster 5 [25]. Clinical evaluations encompassed the number of missing teeth, mouth opening, mandibular movement, and occlusion as the primary clinical outcome measures. Secondary clinical outcome measures included subjective ailments (like pain, discomfort, difficulties in eating, numbness, and adverse scarring) and objectifiable complications (such as facial nerve palsy and salivary fistula). During follow-up visits, radiographic imaging was conducted whenever feasible to ensure proper bone healing and to check for mechanical issues.

A calliper measured mandibular movements and the maximum interincisal distance. Measurements were documented in millimetres. A metalised polyester foil (Arti-Fol^®^ 8 μm, Dr Jean Bausch GmbH & Co. KG, Cologne, Germany) was placed between all opposing pairs of teeth to evaluate occlusion. The occlusion was considered adequate if all opposing tooth pairs held the foil behind the canines. In contrast, the occlusion was considered inadequate if the foil could be pulled out without resistance.

### Radiological examination

Cone-beam computed tomography (CBCT) controlled the reduction intraoperatively or immediately postoperatively. In the operating theatre, this was done by the xCAT^®^ ENT from Xoran Technologies LLC (Ann Arbor, MI 40108, USA). Postoperatively, three-dimensional imaging was obtained with the 3D Accuitomo F8 from J. Morita Europe GmbH (Dietzenbach, Germany) with a maximum field of view of 80 × 80 mm. Further radiographic control imaging took place during follow-up. DICOM data were extracted and further processed.

**Fig. 3:**
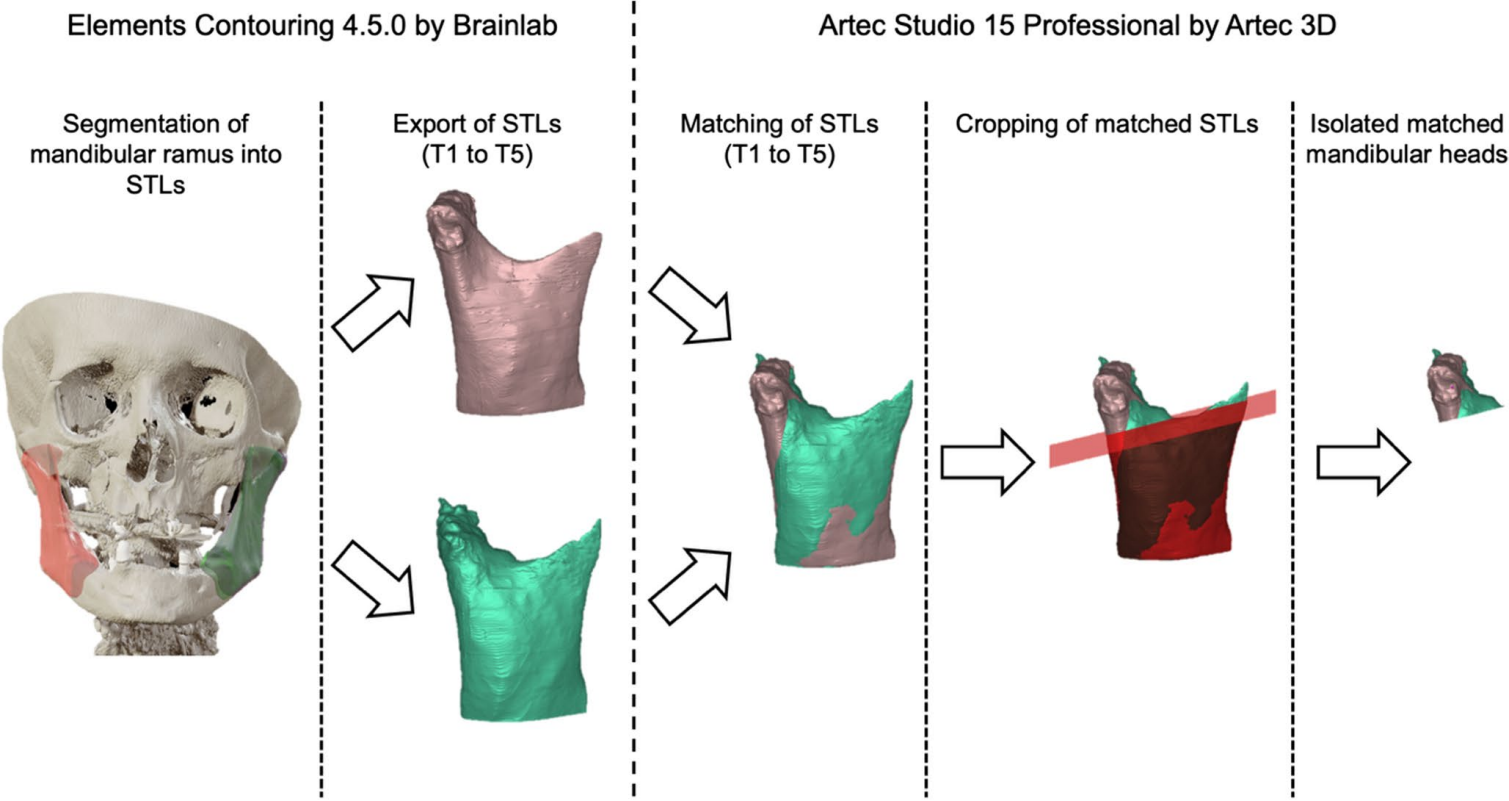
Flowchart of the workflow to obtain three-dimensional data. The graph provides an exemplary overview of the steps to create segmentations and prepare them for further analysis by matching and cropping. Further steps are depicted in Fig. 4

The radiographic images were manually segmented without the aid of any algorithm or artificial intelligence using the workflow Elements Contouring 4.5.0 by Brainlab (Brainlab AG, Olof-Palme-Strasse 9, 81829 Munich, Germany) to determine the temporomandibular joint process volumes through the extracted DICOM datasets. The volumes were further processed in the Artec Studio 15 Professional x64 program (version 15.1.2.60) from Artec 3D (Artec 3D, 20 rue des Peupliers, L-2328, Luxembourg). The segments were now ideally superimposed in the ramus region, excluding the mandibular condyle during the “matching” process. Subsequently, the segments were “cropped” through the A-line or Loukota-line, which runs orthogonally to the posterior ramus line (baseline or also vertical ramus tangent) on the mandible, through the most caudal point of the incisura semilunaris [27]. The volumes created comprised the entire condylar head and a small portion of the neck (Fig. 3). For each time point, the volumes and surfaces of the created segments were recorded.

**Fig. 4:**
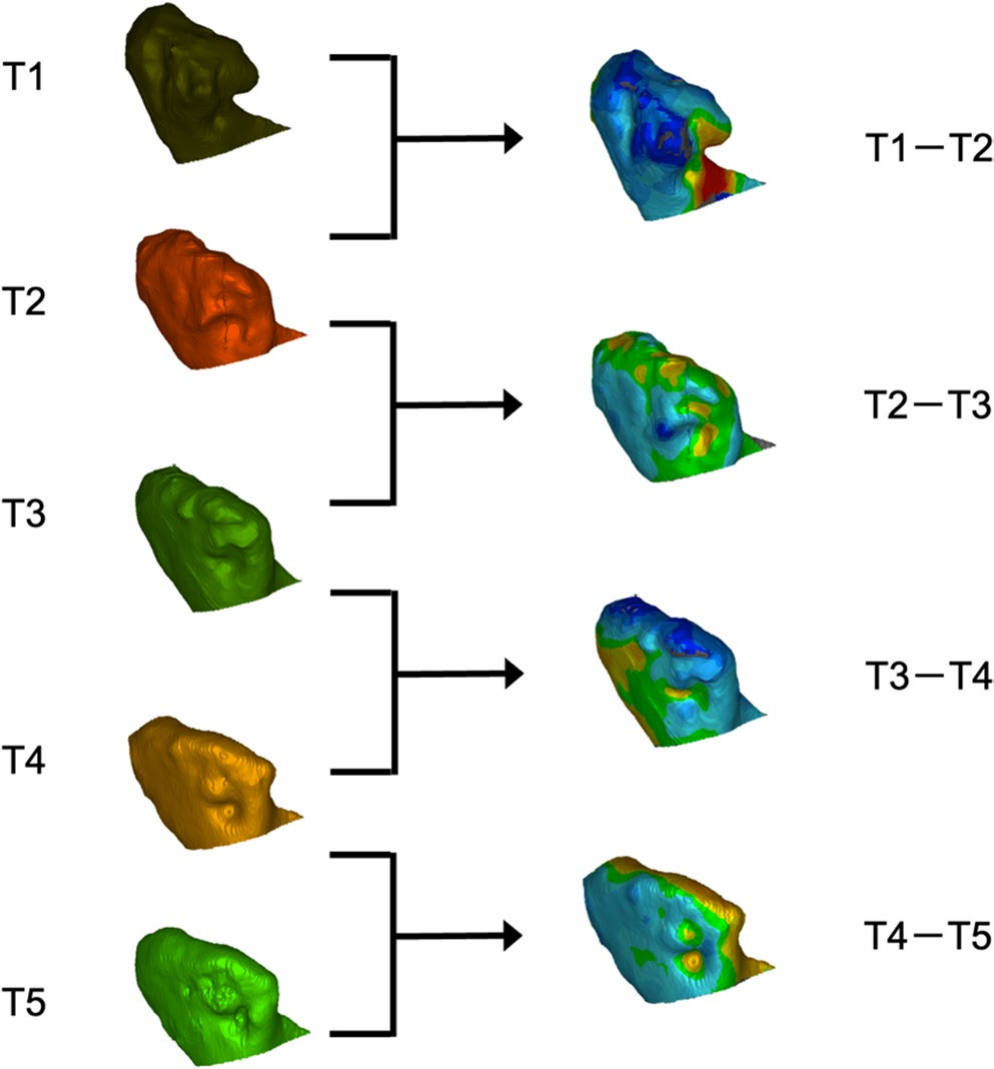
On the left side, the cropped mandibular condyles are seen as recorded for the various Clusters (T1 through T5). In the program, the segments are matched as presented in Fig. 2. The right side depicts the heatmaps of the compared clusters. The colour coding is as follows: green represents a perfect match with 0 mm discrepancy, while red represents a positive value, and blue represents a negative value relative to the segment in comparison

The segments from the different examination time points were compared using the parameter root mean square error (RMS) to quantify the changes in the virtual three-dimensional models. The RMS is calculated by covering the two segmented bodies with point clouds. The distances between these paired point clouds are then measured. The measured values are squared and subsequently square-rooted, resulting in positive values. The smaller the value, the higher the matching of the two bodies, verifying ideal congruence. The larger the values, the greater the degree of incongruence. The computed values were given in millimetres, and for easier identification of where the spatial differences are located, so-called heatmaps were applied to the segmented model to increase visualisation (Fig. 4). For further reading, a supplementary file is available with a detailed, step-by-step description of the method applied (Supplementary File 1).

#### Statistical analysis

Intra- and interobserver bias was excluded by employing repeated measures for digital volume tomograms of a randomly selected subject in our database at two different points in time by two different investigators. The intraclass correlation coefficient (ICC) form selected was the two-way random-effects model analysis of variance (ANOVA), single rater type, checking for absolute agreement [28].

In addition to the descriptive evaluation, all data collected were analysed with Prism 10 Version 10.4.1 (GraphPad Software, San Diego, California, USA), documented with standard deviation, and displayed graphically. Upon checking the normal distribution employing the Shapiro-Wilk test, the values of the groups for clinical data and radiographic measurements were compared using appropriate tests, where the significance level was set to α = 0.05. Spearman’s correlation was used to assess the correlation between various parameters.

## Results

### Patients

We included 58 patients, 35 male (60.3%) and 23 female (39.7%), a ratio of 1.5 to 1. The overall mean age was 46.1 ± 18.0 (95% CI 41.3 to 50.8), ranging from 17 to 87 years. On average, male patients were 43.2 ± 17.9 (95% CI 37.0 to 49.3), and female patients were 50.5 ± 17.6 (95% CI 42.9 to 58.1) years old, which was not statistically different (Mann-Whitney test; *p* = 0.0944, U = 297).

Reasons for hospitalization were falls in 33 (56.9%), followed by road traffic accidents in 18 (31.0%), assault in 5 (8.6%), and leisure accidents in 2 (2.5%) individuals.

### Fractures

The 58 patients suffered from 78 condylar head fractures, meaning that 20 (34.5%) patients had bilateral condylar head fractures. We observed 35 fractures affecting the right side (44.9%) and 43 fractures affecting the left side (55.1%). The exact distribution of fractures according to AOCMF is shown in Table 1.

**Table 1:** Overview of the fractures treated according to AOCMF classification system [10]

	AO Level 3	Amount (*n*)	Relative (%)
All patients	Hp0	33	42.3
	Hp1	34	43.6
	Hp2	8	10.3
	Hm0	3	3.8
	Total	78	100.0

### Follow up rate

The overall response rate of patients was 75.9% in Cluster 1, 75.9% in Cluster 2, 69.0% in Cluster 3, 51.7% in Cluster 4 and 48.3% in Cluster 5. The long-term follow-up analysis in Cluster 5 ranged from 385 to 1933 days (1.1 to 5.3 years) from surgery to examination. The average long-term observation was 963.6 ± 468.9 (95% CI 770 to 1157) days or 2.6 ± 1.3 (95% CI 2.1 to 3.2) years.

### Clinical data

The average number of missing teeth was 5.7 ± 7.7 (median 3.0, 95% CI of the mean 2.6–7.7). We found satisfactory occlusions in 90.9% of patients in Cluster 1, 88.6% in Cluster 2, 97.5% in Cluster 3, 93,3% in Cluster 4, and 100% in Cluster 5.

**Fig. 5:**
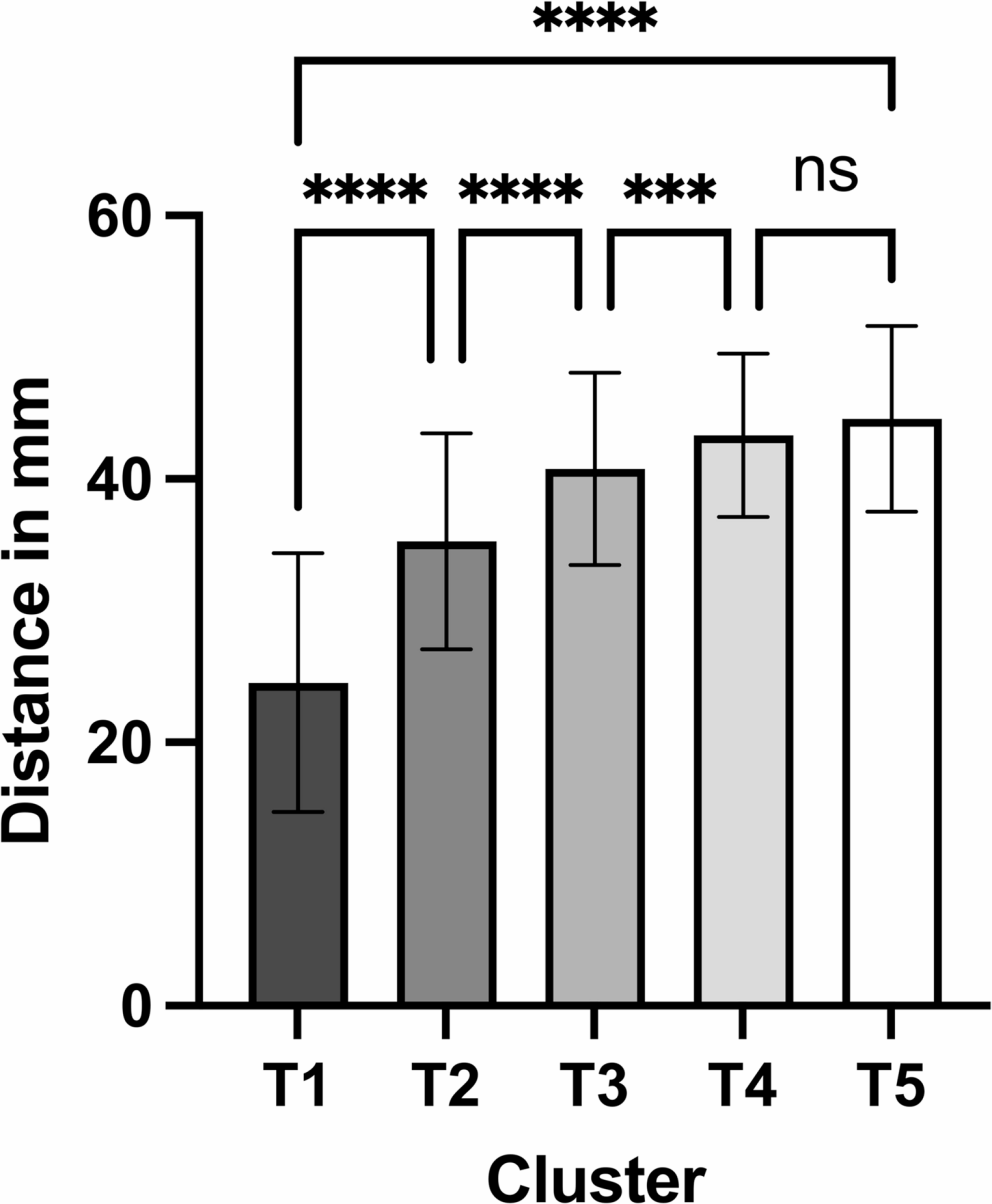
Measured maximum mouth opening: the bars represent the mean, the whiskers depict the standard deviation; significances are drawn from Tukey’s multiple comparisons test (**** = *p* < 0.0001; *** = *p* < 0.001; ns = not significant)

During the different examination intervals, we observed a statistically significant improvement in mouth opening (Fig. 5) and mandibular excursions, i.e., laterotrusion and protrusion (Tables 2 and 3). There were no significant differences in lateral excursions between the left and right sides.

**Table 2:** Clinical parameters: mouth opening (maximum interincisal distance) and protrusion

Clinical parameter	Mean ± SD	95% CI of mean
Mouth opening		
Cluster 1	24.6 ± 9.8	21.5 to 27.6
Cluster 2	35.3 ± 8.2	32.8 to 37.8
Cluster 3	40.7 ± 7.3	38.4 to 43.1
Cluster 4	43.3 ± 6.2	41.1 to 45.6
Cluster 5	44.6 ± 7.1	41.8 to 47.3
ANOVA p-value	F = 94.780, df = 1.547, < 0.0001^†^	
^†^Mixed-effects analysis		
Protrusion		
Cluster 1	2.0 ± 1.8	1.5 to 2.6
Cluster 2	5.4 ± 3.1	4.5 to 6.4
Cluster 3	7.7 ± 2.6	6.8 to 8.5
Cluster 4	8.0 ± 2.7	7.0 to 9.0
Cluster 5	7.6 ± 2.5	6.6 to 8.5
ANOVA p-value	F = 47.642, df = 3.393, < 0.0001^†^	

^†^Mixed-effects analysis

**Table 3:** Lateral excursion of the mandible to the left and right sides in all patients

	Laterotrusion right		Laterotrusion left		Paired t-test *p*-value	Mean of differences ± SD; 95% CI
	Mean ± SD	95% CI	Mean ± SD	95% CI		
Cluster 1	2.8 ± 2.9	1.9 to 3.7	2.8 ± 2.8	1.9 to 3.7	> 0.9999	0.0 ± 2.3; −0.7 to 0.7
Cluster 2	6.6 ± 3.8	5.4 to 7.8	6.3 ± 3.1	5.3 to 7.2	0.6024	0.3 ± 4.0; −0.9 to 1.5
Cluster 3	8.5 ± 3.3	7.4 to 9.5	8.2 ± 3.3	7.2 to 9.2	0.5061	0.3 ± 2.6; −0.6 to 1.1
Cluster 4	8.5 ± 3.9	7.1 to 10.0	8.8 ± 3.5	7.5 to 10.1	0.5642	−0.3 ± 2.5; −1.2 to 0.7
Cluster 5	8.7 ± 2.7	7.7 to 9.8	9.4 ± 4.0	7.8 to 11.0	0.2846	−0.7 ± 3.3; −2.0 to 0.6
ANOVA p-value	F = 32.503, df = 2.978 < 0.0001^†^		F = 39.406, df = 2.711, < 0.0001^†^			

^†^Mixed-effects analysis

We recorded transient facial nerve paresis in seven patients (12.1%) during follow-up examinations, which resolved in every patient at the final examination date. There were no salivary fistulae. In Cluster 5, we recorded signs of craniomandibular dysfunction in four patients (6.9%), and five patients (8.6%) complained of hypo- or dysaesthesia of the preauricular skin. Furthermore, two patients (3.4%) showed excessive scarring in the area of the temporal relief cut at the anterior part of the auricular helix. None of the patients reported any subjective alterations in well-being. There were no clinical signs of soft tissue emphysema, nor could an instability of the bone integrity be identified. No patient required revision surgery or implant removal.

### Radiographic data

#### Intra- and interobserver bias

There was an almost perfect agreement for the repeated measurements between and within the two examiners at the two independent points of time for the volume (ICC = 0.81), the surface area (ICC = 0.83), and the RMS (ICC = 0.83).

### Volume

**Fig. 6:**
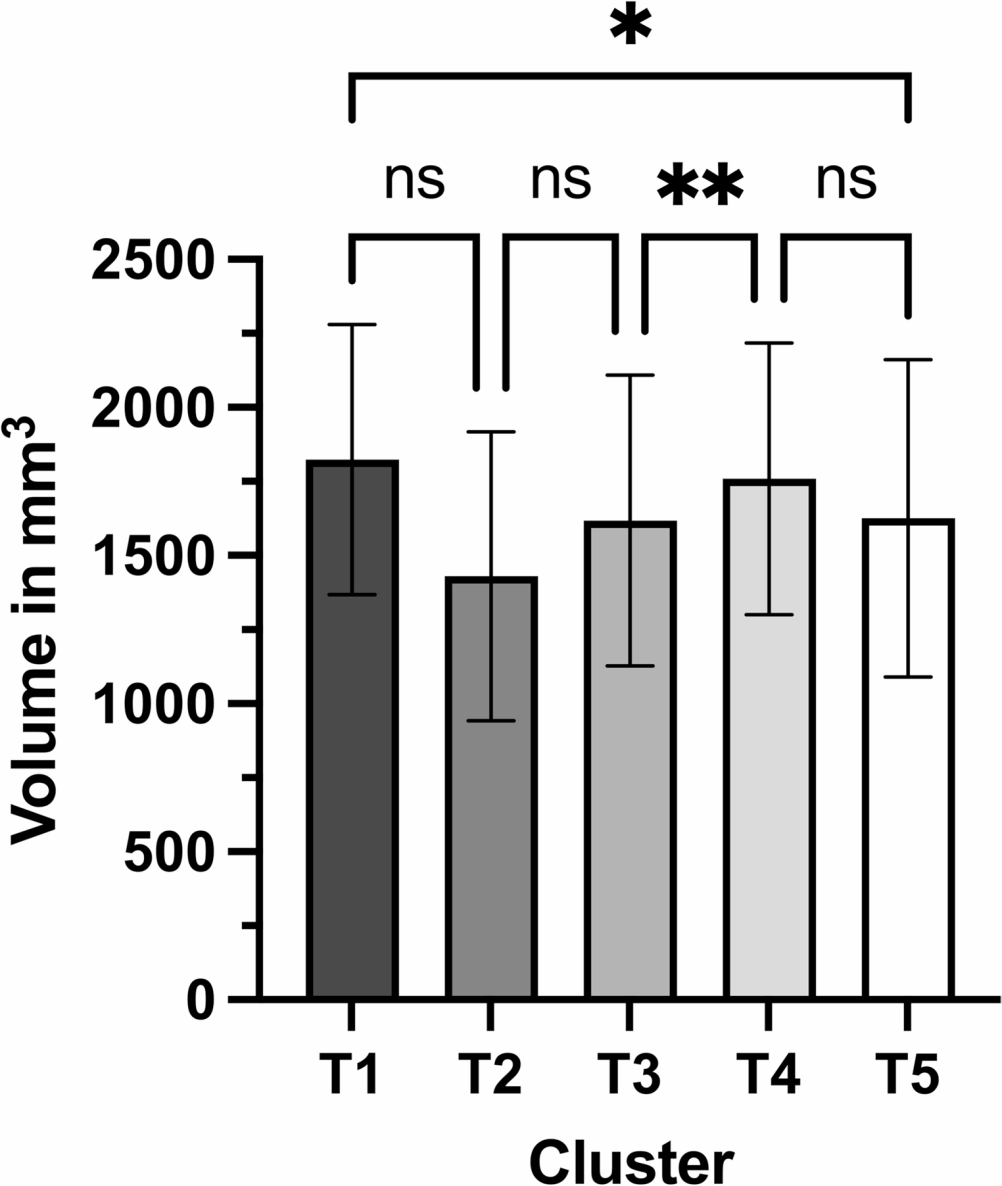
Measured volumes of the healing condylar head: the bars represent the mean values and the whiskers depict the standard deviation; significances are drawn from Tukey’s multiple comparisons test (** = *p* < 0.01; * = *p* < 0.05; ns = not significant)

Initially, we observed a reduction of the condylar volume during Cluster 2, which then gradually increased until Cluster 4 and dropped again in Cluster 5 (Fig. 6; Table 4). Mixed-effects analysis showed no statistically significant changes (*p* = 0.1024). However, the groups had several significant differences when applying Tukey’s multiple comparisons test. Until Cluster 3, there were no significant changes in the volume of the condylar head. Only after Cluster 3, there was a significant increase in Cluster 4 (*p* = 0.0081), followed by an insignificant change in Cluster 5 (*p* = 0.1065). Most importantly, there was a significant reduction in volume between the first and last Cluster (*p* = 0.0267).

The volumes experienced both increases and decreases relative to the initial situation. The overall relative change in volume was 21.18 ± 21.69% (median 14.51%; 95% CI of mean 13.08 to 29.28%).

**Table 4:** Measured volumes of the healing condylar head at various points in time

Volume of the condylar head (in mm^3^)		
	**Mean** ± **SD**	**95% CI of mean**
Cluster 1	1824.4 ± 456.3	1682.2 to 1966.5
Cluster 2	1429.9 ± 487.7	978.8 to 1880.9
Cluster 3	1618.6 ± 490.8	1431.9 to 1805.3
Cluster 4	1758.8 ± 458.7	1573.5 to 1944.0
Cluster 5	1626.1 ± 536.2	1425.8 to 1826.3
Mixed-effects analysis	F = 3.221, df = 0.652, *p* = 0.1024	

### Surface area

**Fig. 7:**
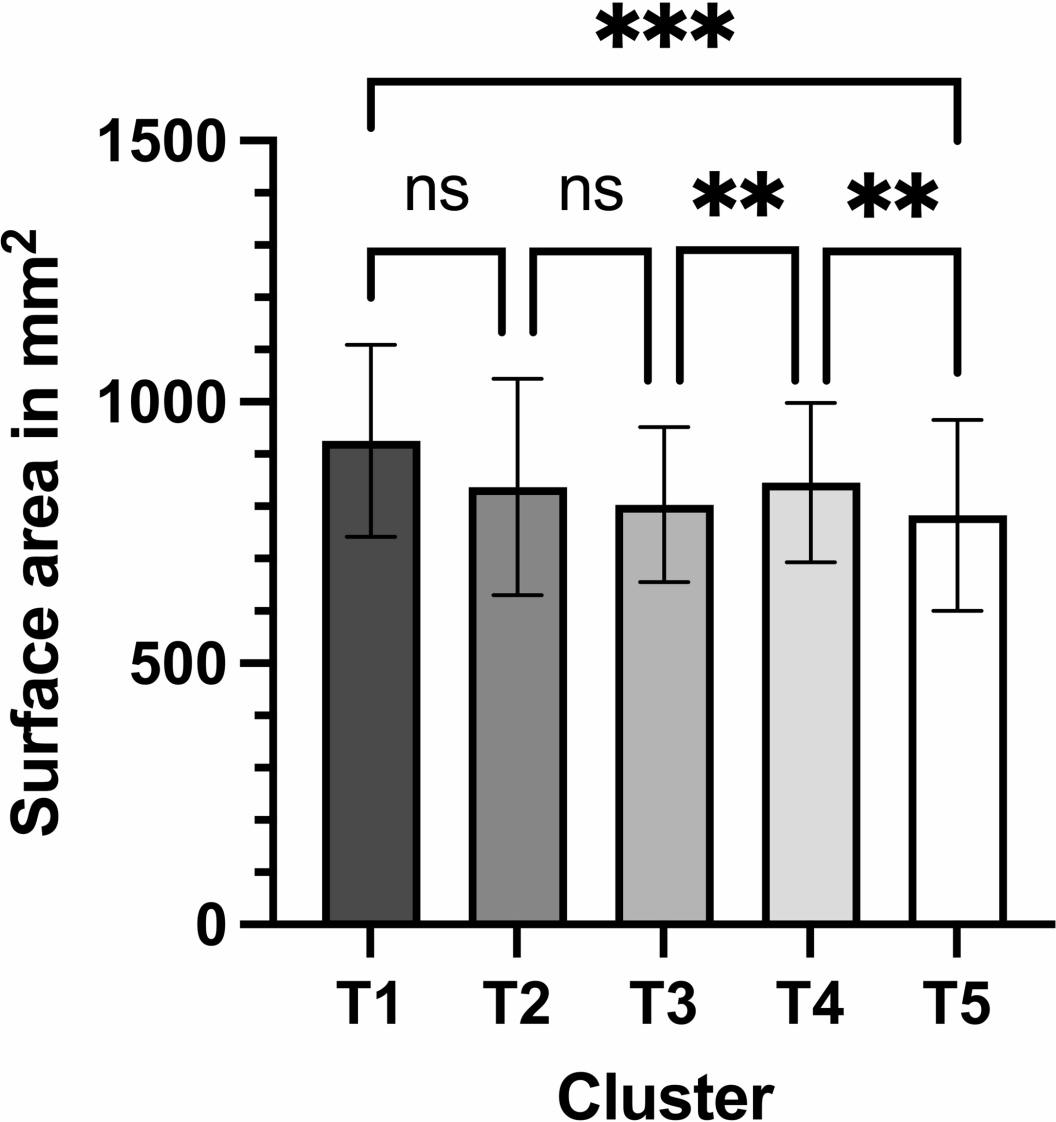
Measured surface areas of the healing condylar head: the bars represent the mean values and the whiskers depict the standard deviation; significances are drawn from Tukey’s multiple comparisons test (*** = *p* < 0.001; ** = *p* < 0.01; ns = not significant)

We observed a continuous reduction in the surface area of the condylar head until Cluster 3, followed by an increase in Cluster 4 and a final drop in Cluster 5. Significant differences (*p* = 0.0073) in surface area over time were computed by mixed-effects analysis. Tukey’s multiple comparisons identified significant changes in the surface area of the condylar head between Clusters 3 and 4 (*p* = 0.0031), 4 and 5 (*p* = 0.0058), and 1 and 5 (*p* = 0.0004) (Fig. 7; Table 5).

**Table 5:** Measured surface areas of the healing condylar head at various points in time

Surface are of the condylar head (in mm^2^)		
	**Mean** ± **SD**	**95% CI of mean**
Cluster 1	925.3 ± 183.6	868.0 to 982.5
Cluster 2	837.1 ± 207.0	645.7 to 1028.5
Cluster 3	803.7 ± 147.7	747.5 to 859.9
Cluster 4	845.3 ± 152.8	783.6 to 907.1
Cluster 5	783.0 ± 182.1	715.0 to 851.0
Mixed-effects analysis	F = 13.733, df = 0.577, *p* = 0.0073^†^	

Similar to the volume findings, the surface areas exhibited increases and decreases relative to the initial situation. The overall relative change in surface area was 21.30 ± 18.68% (median 14.05%; 95% CI 14.32 to 28.27%).

### Root mean square error

Significant differences were found in the surfaces of the condylar heads over time (mixed effects analysis, *p* < 0.0001). The most considerable differences between the surfaces of the condylar head were seen when comparing Cluster 1 versus Cluster 2, which were significant compared to Cluster 2 versus Cluster 3 (Tukey’s multiple comparisons test; *p* = 0.0228). All successive values dropped in magnitude as time passed between ORIF and the radiographic examination, leading to a hyperbolic curve shape. Tukey’s multiple comparisons test identified a statistically significant difference (Tukey’s multiple comparisons test; *p* = 0.0108) between the last two comparisons, even though the absolute difference was minute, measuring 0.295 mm.

**Fig. 8:**
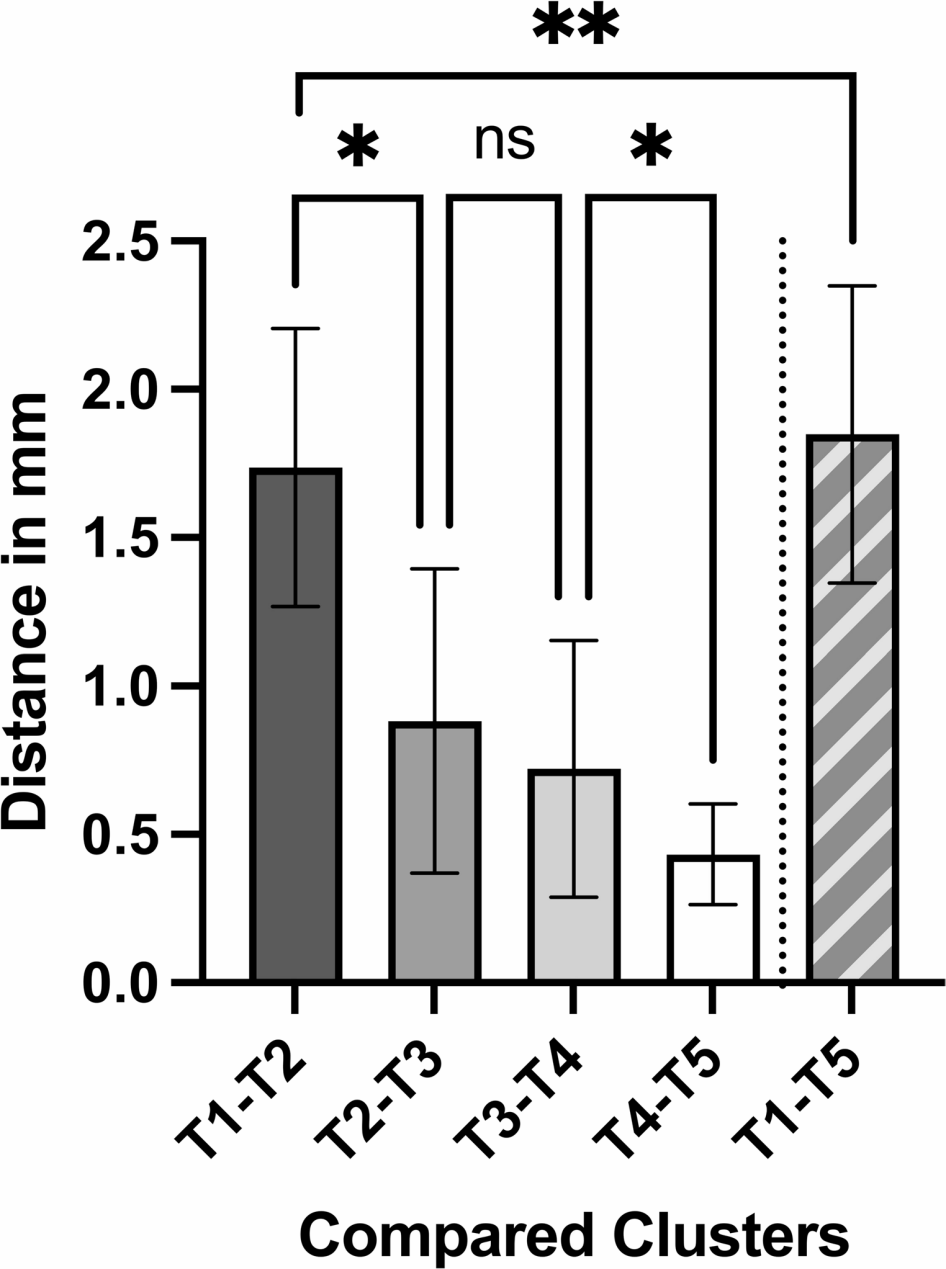
Measured RMS of the healing condylar head: the bars represent the mean values and the whiskers depict the standard deviation; significances are drawn from Tukey’s multiple comparisons test (** = *p* < 0.01; * = *p* < 0.05; ns = not significant)

In order to determine the most relevant changes regarding the remodelling process of the condylar head, the surfaces of the condylar head of Cluster 1 versus 5 were also compared. In absolute numbers, this RMS value had the highest magnitude. Comparing the differences in RMS between Clusters 1 versus 2, and 1 versus 5, minute changes were averaging 0.117 mm, albeit statistically significant (paired t-test, *p* = 0.0057; mean of differences − 0.117 ± 0.232 (95% CI −0.198 to −0.037) mm) (Fig. 8; Table 6).

**Table 6:** RMS of the healing condylar head

Root mean square error of the condylar head (in mm)		
	**Mean** ± **SD**	**95% CI of mean**
Cluster 1 vs. 2	1.736 ± 0.469	1.582 to 1.890
Cluster 2 vs. 3	0.882 ± 0.512	0.610 to 1.155
Cluster 3 vs. 4	0.721 ± 0.432	0.513 to 0.930
Cluster 4 vs. 5	0.433 ± 0.170	0.339 to 0.527
Cluster 1 vs. 5	1.849 ± 0.501	1.684 to 2.013
ANOVA p-value	F = 49.742, df = 2.157, < 0.0001^†^	
^†^Mixed-effects analysis		

### Miscellaneous

A radiographic phenomenon occurred approximately 3 to 9 months after osteosynthesis, presenting as irregular radiolucent cavities around the Mg-CS. These observed “gas lacunae”, however, showed a complete remission at the Cluster 5 follow-up radiograph.

None of the magnesium screws became exposed or protruded from the condylar surface during follow-up.

Spearman’s correlation was used to assess the correlation between various parameters. No statistically significant correlation was identified between RMS, relative and absolute change in volume and surface area, and clinical parameters such as missing teeth, protrusion, laterotrusion, and maximum mouth opening, and the patient’s age or gender.

## Discussion

### Key results

The ORIF of CHF employing a Mg-CS showed a significant loss in volume and surface area of the condylar head during the healing process. The work employed quantitative measurements for three-dimensional changes already used for computer-assisted design and manufacturing in the industry. There were changes in three-dimensional congruence, which were significant during the first 100 days, or three months, after ORIF. Despite these remodelling processes, there were excellent clinical results with a low complication rate, rendering ORIF with Mg-CS an excellent treatment modality for CHF.

### Limitations

The study’s main limitation was its retrospective design without randomisation or a control group. Despite the high-resolution pictures of the CBCT, the field of view was limited and did not include the contralateral condylar head, thereby excluding comparison with the healthy, unaffected side. No extra scan was performed of the healthy side for reasons of radiation hygiene.

### Interpretation

Increasingly, ORIF is the treatment of choice for CHF, with recent meta-analyses and systematic reviews concluding that this produces superior functional outcomes to conservative treatment [29]. However, opinions remain divided regarding the choice of osteosynthesis material [9]. The unique configuration of the condylar head imposes significant mechanical demands on the osteosynthesis material [1, 9]. One possibility to fix small bones or small fragments to larger bones is the use of cannulated compression screws. The technique proved reliable and easy to perform in general traumatology [30, 31] and was initially introduced into maxillofacial surgery in 2007 for CHF [32]. Other locations for application within the mandible followed, such as the anterior body [33] and the mandibular angle [34]. The surgical technique of using a titanium Herbert screw for CHF disappeared from the literature until recently [25].

Although some authors promote the removal of titanium osteosynthesis placed in the condylar head [35, 36], most only advocate doing so when there is evidence of material loosening or unfavourable remodelling such that the material becomes exposed [14, 37]. As a result, biodegradable fixation has been proposed using polylactic-acid-based materials [38] and biodegradable magnesium alloys [22]. Thus far, long-term results for osteosynthesis using biodegradable magnesium alloys have not yet been published, and the presented work accounts for a maximum follow-up of five years and three months.

The extended follow-up is crucial for biodegradable materials as the material degradation process could undoubtedly induce physiological changes within the condylar head bone, which may adversely affect healing or bone turnover, induce unfavourable remodelling, or negatively impact the surrounding soft tissues.

Magnesium is highly susceptible to corrosion, especially in aqueous environments. The degradation process primarily involves the anodic dissolution of magnesium, leading to the formation of magnesium ions (Mg²⁺) and the cathodic evolution of hydrogen gas (H₂) [39]. Histological analysis revealed that implant degradation began within 1 week, progressing to approximately 50% degradation by 12 weeks. Moderate bone apposition was noted around the implant during this period. By 52 weeks, the metallic component was fully degraded [40]. Therefore, follow-up until and beyond this time is crucial. There were radiographic signs of periimplant gas formation in some patients in Clusters 3 and 4 that were no longer present in Cluster 5. This finding is in concordance with those of other authors [41, 42]. Rather than being purely detrimental, low-level hydrogen formation appears to stimulate periosteal cell proliferation, modulate the microenvironment, and enhance osseointegration. Thus, hydrogen is portrayed as a physiologically supportive factor rather than a toxic byproduct [43]. Closing the circle on the clinical picture presented by the examined patients within the study, adverse effects connected to gas formation within the tissues or any subjective alterations in well-being were reported by none of the patients. Additionally, the clinical parameters did not show any detrimental signs or impaired function. The contrary is true – there are well-documented signs of adequate reconstitution of mandibular excursion in all patients, as discussed in the following paragraph.

As is expected in the recovery from ORIF of CHF, there was a gradual improvement in mouth opening and mandibular excursions. Normal mandibular excursion, in particular mouth opening expressed as the interincisal distance, is crucial for adequate masticatory function and, thus, food intake. Literature reports interincisal distances ranging from 37.8 ± 4.2 to 50.5 ± 5.1 mm [18, 20, 23, 36, 44], aligning with our study’s findings. Exploring the lateral and anterior mandibular movement after ORIF of CHF is less common. However, available studies [18, 23, 37], including ours, indicate satisfactory results in any mandibular excursion. No statistical differences were identified when comparing left and right laterotrusion. Adequate stability of osteosynthesis is crucial for rapid recovery [45]. Therefore, we conclude that the condylar head received stable ORIF and that function was satisfactorily restored compared to the contralateral side.

Besides the mandibular excursion, restoration of normal occlusion following mandibular fracture treatment is the second factor required for adequate masticatory function. The reported rates of occlusal disturbances after ORIF range from 0 to 10% [14, 20, 23, 44, 46]. Our study found no permanent occlusal disturbances at the final examination, thus concurring with the existing literature.

Facial nerve palsy is a detrimental complication of surgical treatment for CHF. The literature varies widely on rates of temporary and permanent facial paralysis [14, 18, 20, 46, 47]. Notably, persistent facial nerve palsy was predominantly associated with implant removal procedures, possibly due to tissue scarring and nerve dissection difficulties [48, 49]. However, our study employed biodegradable alloys for osteosynthesis, rendering implant removal obsolete, ultimately leading to the absence of facial paralysis in Cluster 5.

Since 2010, three-dimensional analysis of the condylar head has been studied [50]. While some studies attempt to describe the volume of the condylar head, absolute values are often not reported. Instead, volume is expressed relative to the total volume of the entire mandible [51] or ramus [52]. Specific research focuses on the volume of the condylar head in the context of traumatology or surgical treatment of the mandibular condyle and head itself [25, 35, 36, 44, 53]. While some studies only report postoperative volume loss without absolute values [36], others provide statements on absolute values at various examination times [25, 35, 44, 53]. These absolute values are higher than those measured in the current study. The greater volume in the other studies could be explained by different measurement methods, i.e., differences in the cutting planes used to segment the condylar heads [44, 53]. However, despite lower absolute volume changes in the present study compared to the literature, the relative differences in volume change (ranging from 15.29% to 16.0%) are distinctly more eminent. A possible explanation could be that the presented study worked with biodegradable implants, which aim to provoke the body’s response to the implant, which titanium, due to its bioinert properties, does not. One study utilized the same ORIF and examination methods and found approximately the same absolute changes, albeit increases in the volume of the condylar head. Interestingly, the relative changes were lower compared to the ones in the presented study. However, titanium alloys were used for ORIF [25]. In addition, the initial condylar volume of the patients in the presented study was smaller, and the volume of the Mg-CS implant inserted was also more substantial relative to the condylar volume. The implant could have exerted a more considerable effect on the reaction of the patient’s body to the implant during the degradation process, causing the more pronounced changes in volume observed in the study population.

Likewise, only a few reports in the literature examine the surface area of the condylar head. One analysis focuses on the surface area of the condylar head, but the cut-off plane used to define the condylar head is set further cranially than in the current study [50]. Another group investigating structural changes in the condylar head after ORIF attempts to measure the change in the surface of the joint by assessing the distance between the osteosynthesis screws and the joint surface using three-dimensional data. However, this results in a two-dimensional analysis in three planes without determining the actual surface of the condylar head, making comparisons with the presented results impossible [37]. Finally, some reports employed the same data acquisition technique and measured lower absolute and relative values for the surface area of the condylar head employing a titanium headless compression screw [25]. Again, this could be explained by the different biological inertness and degradability of the implants used.

Further, our study demonstrated a reduction in the surface area of the condylar head. This change in surface area may be due to the remodelling process of the healing condylar head, where the initially high surface area due to indentations and protrusions of the individual bone fragments is filled by the body, thereby smoothing the protrusions and indentations, resulting in a smaller surface area. The surface volume change could be considered an indicator of three-dimensional change.

Some scientific reports have already used the RMS to ascertain the symmetry of the mandibular ramus [52]. A novel approach to objectively determine three-dimensional change using the RMS was first described recently. The computed values were only between two CBCT scans, did not reveal actual plasticity but showed a definite change in the surface configuration, and came up to 1.6 mm [25]. The presented study was able to distinguish the time frame of the most significant changes in the healing condylar head, which was during the first hundred days, or three months, after ORIF. This distinguishment was confirmed by comparing the changes between the first two CBCT scans and the first and last CBCT scans, reaching up to 1.7 and 1.8 mm, respectively. There were significant differences between these RMS values. One current publication confirms the most pronounced remodelling changes in the condylar head within the first three months after ORIF [54]. Notably, despite the differences in surface areas and volumes, there was still virtually the exact change between the three-dimensional models over time, regardless of the material used for the osteosynthesis of CHF.

The use of ORIF for CHF remains a topic of debate. However, in this study, all methods employed for treating patients were state-of-the-art, effectively addressing virtually all fracture patterns of the condylar head. Magnesium alloys exhibited higher relative volume and surface area changes than reported in the current literature, which employed titanium implants. The same changes were observed between three-dimensional models at different points in time, without suggesting poorer functional outcomes. The proposed approach to address surface plasticity is a novel method to quantifying three-dimensional changes of the healing condylar head. Unfortunately, the direction and location of surface change can currently only be displayed graphically, not mathematically. Despite this fact, there was still a low incidence of adverse effects, no mechanical complications were noted, and a successful restoration of normal mandibular function was achieved, even in the long-term follow-up of up to five years. The study supports the use of Mg-CS as an appropriate treatment modality for CHF.

## Supplementary Information

Below is the link to the electronic supplementary material.

Supplementary Material 1 (PDF 1.04 MB)

## Data Availability

The datasets generated during and analysed during the current study are available from the corresponding author upon reasonable request.
