# Supplementary material for: Functional and three-dimensional radiographic outcomes after open reduction and internal fixation of condylar head fractures using magnesium alloy cannulated screws – a retrospective long-term follow-up
Source: Clin Oral Investig. 2025 Sep 29;29(10):479. doi: 10.1007/s00784-025-06585-x (PMC12477083; doi:10.1007/s00784-025-06585-x)
Supplement: Supplementary file 1 — Supplementary Material 1 (PDF 1.04 MB) [file 784_2025_6585_MOESM1_ESM.pdf]

Step-by-step guide for the data acquisition of the study:

Functional and three-dimensional radiographic outcomes after open reduction and internal fixation of condylar head fractures using magnesium alloy cannulated screws – a retrospective long-term follow-up

Segmentation in Contouring 4.5.0 by Brainlab

1. Opening of the Remote Desktop Control of the Brainlab User Interface via Origin Server with any recent version of a web browser (Fig. 1).

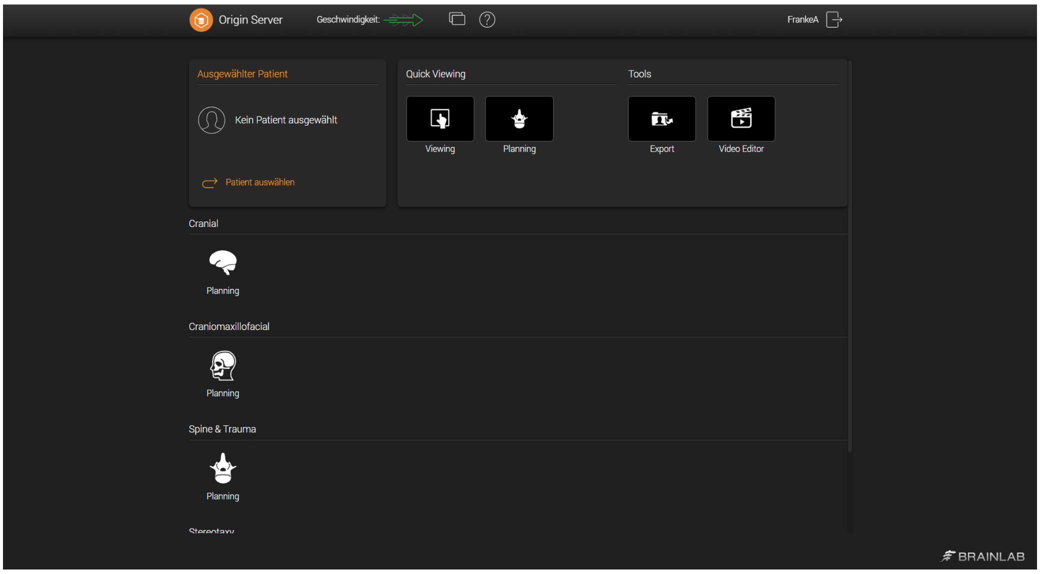

Figure 1 Screenshot of the Origin Server Interface for Brainlab.

2. Activation of the Plugin *Craniomaxillofacial planning* (Fig. 2)

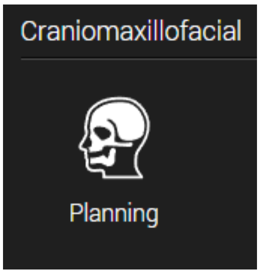

Figure 2 *Craniomaxillofacial planning* button within the Origin Server Interface.

3. Selection of the DICOM data set for the patient of interest.
4. Entering the segmentation menu: *Planning* → *Object Management*

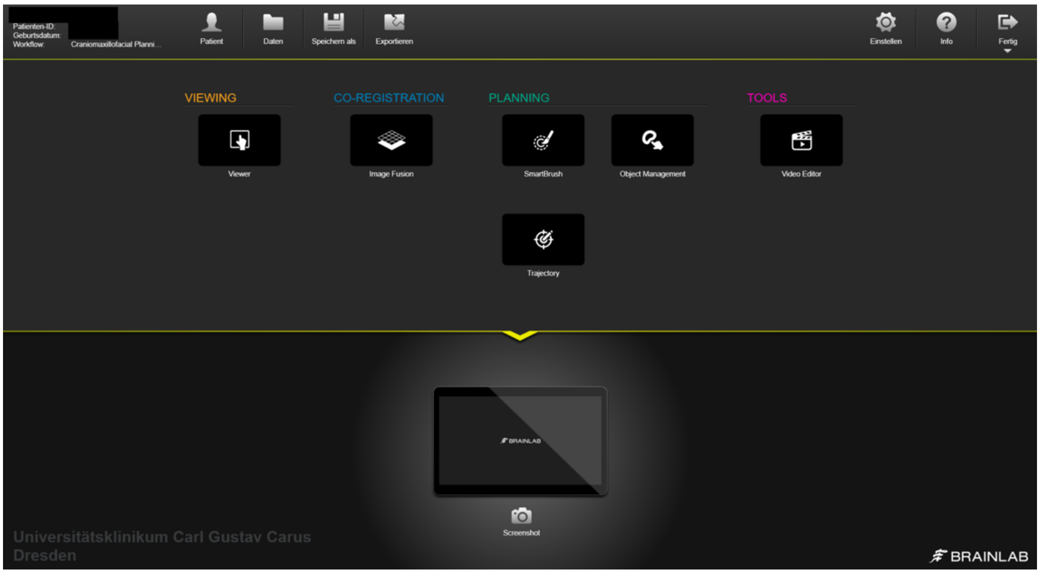

Figure 3 Screenshot of the Craniomaxillofacial Planning Interface.

5. Through the *Contouring* subsection, the region of interest, i.e. the entirety of the mandibular ramus, is segmented in the axial, coronal and sagittal views using the *SmartBrush* tool. After finishing the contouring or segmentation, the *Craniomaxillofacial planning* window is reaccessed by pressing *Done*.

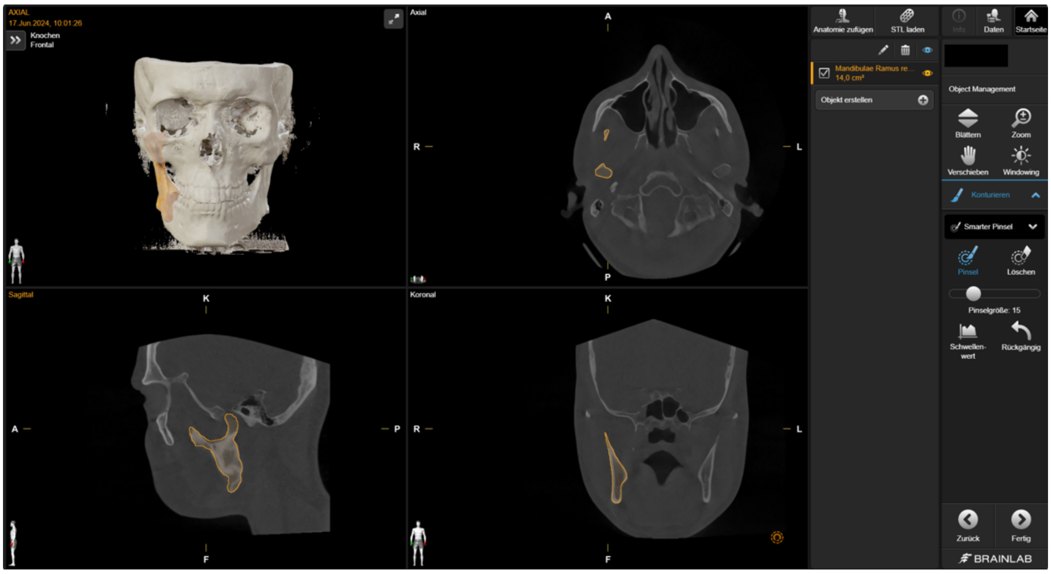

Figure 4 Screenshot of the Object Management Interface. A segmentation of the right mandibular ramus is in progress.

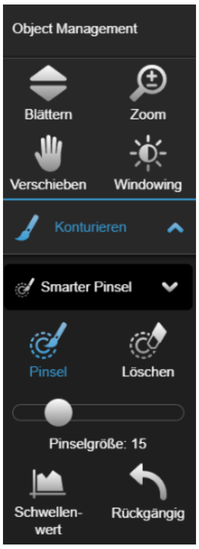

Figure 5 Detail view of the Object Management Tool with the subsection *Contouring*. The *SmartBrush* tool is activated.

6. The segmented model is exported by selecting the respective segmentations of interest, which will be saved on the hard drive physically located at the central server using the *STL Export* button.

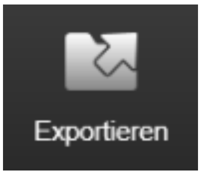

Figure 6 Export button within the Craniomaxillofacial Planning Interface.

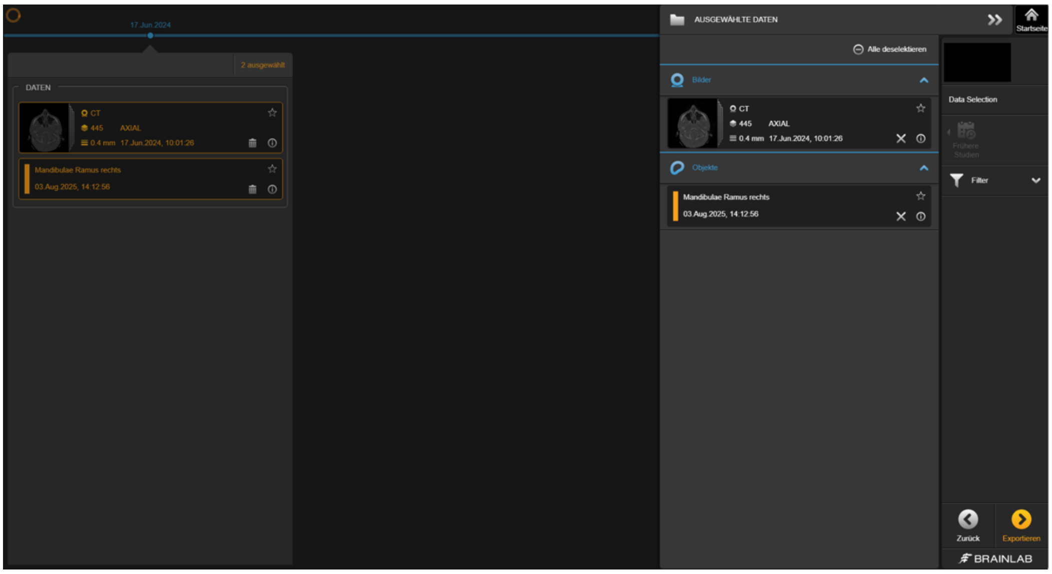

Figure 7 Screenshot of the Export Interface.

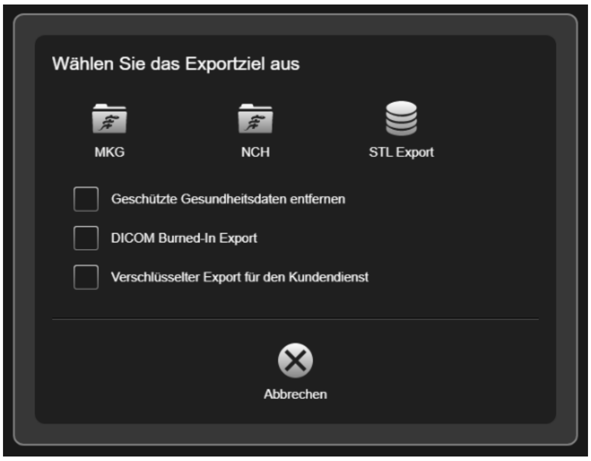

Figure 8 Detail of the possible export modalities.

7. The server is accessed using the ftp view of the browser to select and export the STL files on the local hard drive for further processing.

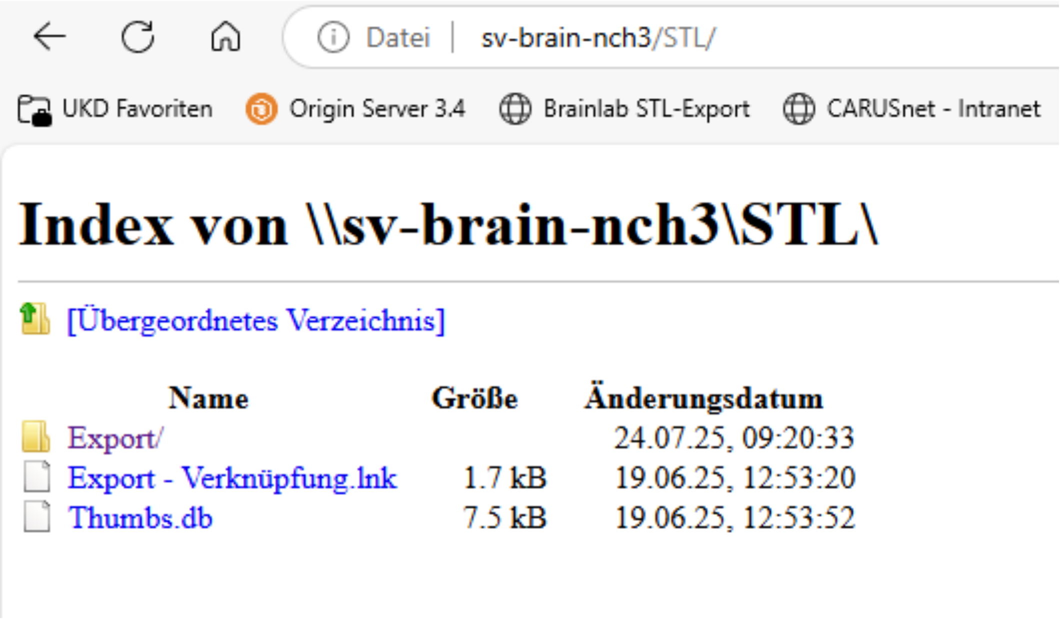

Figure 9 Detail of the ftp view of Microsoft Edge.

Segment (STL) processing in Artec Studio 15 Professional by Artec 3D

1. The software is run on the system.
2. A new project file is created (Shortcut "*Ctrl-N*")
3. The segments (STLs) are imported into the project file (Shortcut "*Ctrl-I*")
4. First, all the imported segments are selected in the *Workspace*. The segments are aligned to the T1 segment using *Align* → *Auto-alignment*.

   If *Auto-alignment* does not allow for adequate superimposition, manual corrections are performed. *Align* → *Rigid (markers)* → *Align markers*. The segment T1 serves as the baseline template, and all other segments are aligned accordingly. At least three stable anatomical landmarks are selected, such as the posterior border of the mandible, the lingula mandibulae and the muscular process. Between the two segments, point pairs are created at the same anatomical landmarks. For the process of superimposition, the actual mandibular condyle is ignored to account for possible changes due to remodelling.

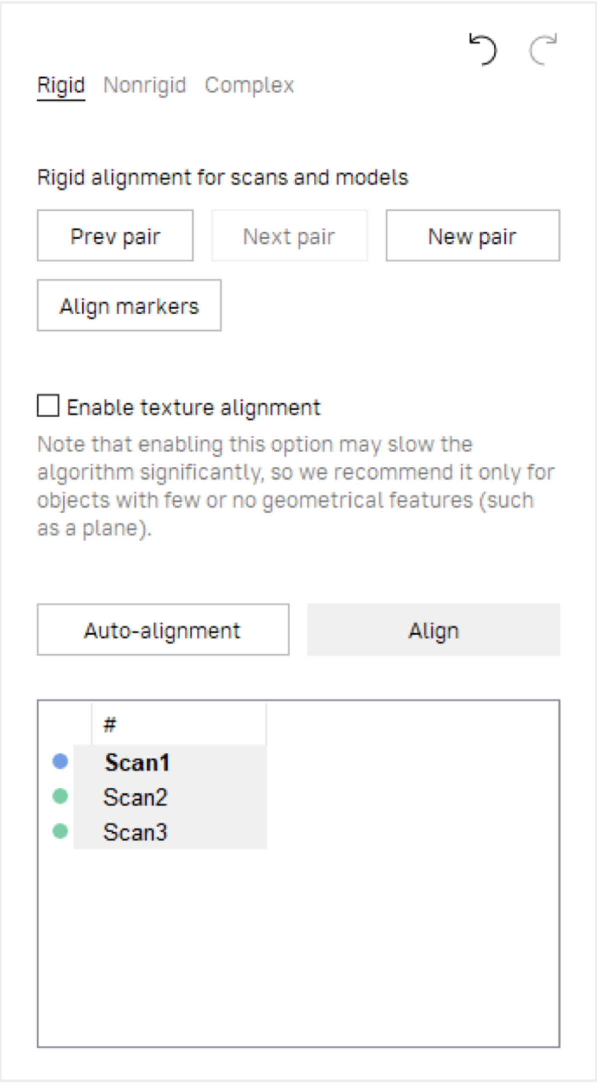

Figure 10 Detail of the *Alignment* modalities.

5. Step 5 is repeated for every single segment (T1-T2, T1-T3, T1-T4, and T1-T5) to ensure perfect alignment of the mandibular ramus except for the mandibular condyle.
6. All the segments are now cropped. Open *Editor* → *Eraser* → *Cutoff-plane selection*. The panel instructions are followed to crop all the segmentation in the same place identified by the A-line. The posterior tangent serves as the primary vector to align the subsequent orthogonal planes that are created.
7. The cropped segments are individually measured for volume and surface area.
   Measures → *Sections and volume* → *Calculate*

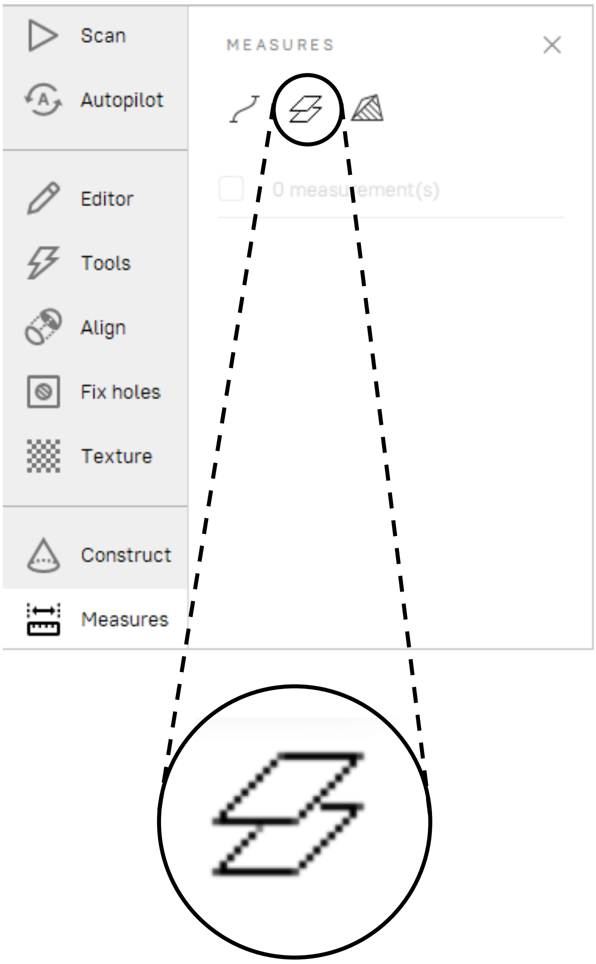

Figure 11 Detail of the *Measures* modalities. Bottom: magnification of the *Sections and volume* tool.

8. Three-dimensional congruence is tested using the *Measures* function, comparing the segments at various time points, i.e., T1-T2, T2-T3, T3-T4, T4-T5, and T1-T5.
   Measures → *Distance map* → *Search distance* → *Calculate*.

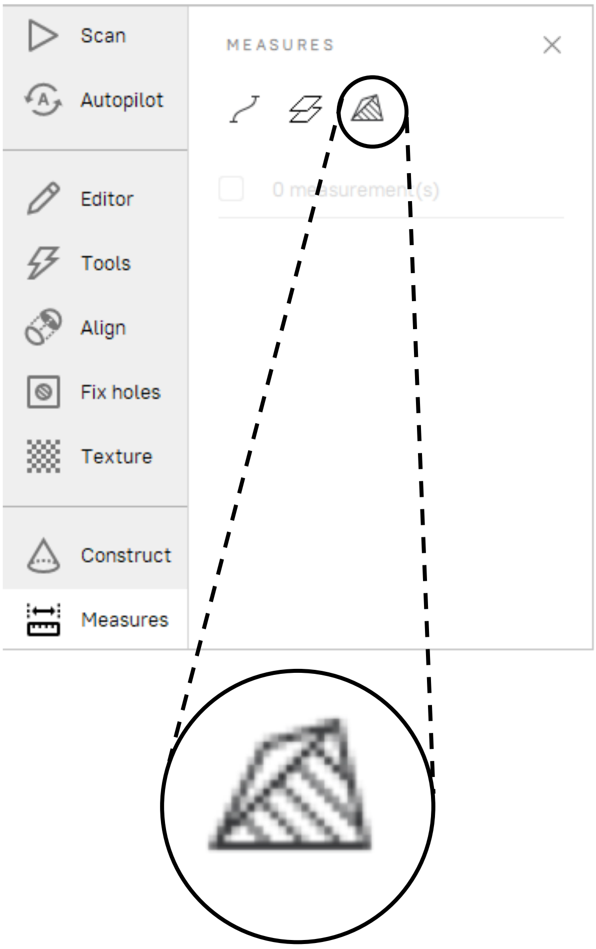

Figure 12 Detail of the *Measures* modalities. Bottom: magnification of the *Search distance* tool.
